# Supplementary material for: Using Behavioural Insights to Improve the Uptake of Services for Drug and Alcohol Misuse
Source: Int J Environ Res Public Health. 2021 Jun 28;18(13):6923. doi: 10.3390/ijerph18136923 (PMC8297083; doi:10.3390/ijerph18136923)
Supplement: Supplementary file 1 [file ijerph-18-06923-s001.zip › ijerph-1197542-supplementary.pdf]

Supplementary File S1: Behavioural Insights Study Survey

Instructions: Please complete the below questions as honestly as possible and then place the questionnaire in the envelope provided and hand in to reception.

If there are any questions you do not wish to complete, please tick the 'prefer not to say' option and move on to the next question.

1. Age: …………. years

2. Gender (please tick)

☐ Male ☐ Female ☐ Transgender
☐ Prefer not to say ☐ Not Applicable

3. Employment status (please tick)

☐ Employed full time ☐ Employed part time ☐ Self-employed
☐ Full time education ☐ Part time education ☐ Unemployed
☐ Prefer not to say ☐ Not Applicable

4. Relationship Status

☐ Married ☐ Cohabiting ☐ In a relationship
☐ Single ☐ Prefer not to say ☐ Not Applicable

5. How many (if any) dependent children do you have?

…………………. (number) ☐ Not Applicable ☐ Prefer not to say

6. Please indicate which (if any) of the below substances you currently use

☐ Alcohol ☐ Tobacco ☐ Marijuana
☐ ecstasy ☐ MDMA ☐ heroin
☐ GHB ☐ Ketamine ☐ PCP
☐ LSD ☐ mushrooms ☐ Anabolic steroids
☐ Poppers ☐ Cocaine ☐ Methamphetamine
☐Benzodiazepines ☐ Amphetamines ☐ Crack Cocaine
☐ Methadone ☐ other opioids ☐ other

☐ other stimulants ☐ Prefer not to say ☐ Not Applicable

7. **Please indicate your treatment status:**

☐ Receiving Treatment at HART ☐ Attending for Assessment ☐ Declined All Treatment
☐ Took up treatment elsewhere ☐ Prefer not to say ☐ Not Applicable

8. **Please use this space to provide us with any comments of feedback about the service especially including factors that influenced or could influence your decision to take up treatment:**

9. Please read the statements below carefully and rate how strongly you agree or disagree with each by circling the appropriate number. If you feel the statement does not apply to you, please tick 'not applicable'. If you don't want to answer, please tick 'prefer not to say'.

**1.**	**It is easy for me to get to and from appointments at the treatment centre**						
1	2	3	4	5	6	7	☐ Not Applicable
Strongly Disagree	Disagree	Disagree a Little	Neither agree nor disagree	Agree a Little	Agree	Strongly Agree	☐ Prefer not to say
**2.**	**It is expensive for me to get to and from appointments at the treatment centre**						
1	2	3	4	5	6	7	☐ Not Applicable
Strongly Disagree	Disagree	Disagree a Little	Neither agree nor disagree	Agree a Little	Agree	Strongly Agree	☐ Prefer not to say
**3.**	**Appointments can/do fit in around the rest of my life**						

1	2	3	4	5	6	7	☐ Not Applicable
Strongly Disagree	Disagree	Disagree a Little	Neither agree nor disagree	Agree a Little	Agree	Strongly Agree	☐ Prefer not to say

4. Treatment and appointments will/do take up a lot of my time

1	2	3	4	5	6	7	☐ Not Applicable
Strongly Disagree	Disagree	Disagree a Little	Neither agree nor disagree	Agree a Little	Agree	Strongly Agree	☐ Prefer not to say

5. Treatment and appointments will/do take up a lot of my energy and effort

1	2	3	4	5	6	7	☐ Not Applicable
Strongly Disagree	Disagree	Disagree a Little	Neither agree nor disagree	Agree a Little	Agree	Strongly Agree	☐ Prefer not to say

6. It is/would be useful to receive text-message reminders the day before appointments

1	2	3	4	5	6	7	☐ Not Applicable
Strongly Disagree	Disagree	Disagree a Little	Neither agree nor disagree	Agree a Little	Agree	Strongly Agree	☐ Prefer not to say

7. It is/would be useful to receive appointment letters from the service

1	2	3	4	5	6	7	☐ Not Applicable
Strongly Disagree	Disagree	Disagree a Little	Neither agree nor disagree	Agree a Little	Agree	Strongly Agree	☐ Prefer not to say

8. If I knew how much each appointment cost to provide I'd be more likely to attend

1	2	3	4	5	6	7	☐ Not Applicable
Strongly Disagree	Disagree	Disagree a Little	Neither agree nor disagree	Agree a Little	Agree	Strongly Agree	☐ Prefer not to say

9. Treatment will be beneficial to me

1	2	3	4	5	6	7	☐ Not Applicable
Strongly Disagree	Disagree	Disagree a Little	Neither agree nor disagree	Agree a Little	Agree	Strongly Agree	☐ Prefer not to say

10. Treatment has been beneficial for other people like me

1	2	3	4	5	6	7	☐ Not Applicable
Strongly Disagree	Disagree	Disagree a Little	Neither agree nor disagree	Agree a Little	Agree	Strongly Agree	☐ Prefer not to say
**11.**	**Treatment will not work for me**						
1	2	3	4	5	6	7	☐ Not Applicable
Strongly Disagree	Disagree	Disagree a Little	Neither agree nor disagree	Agree a Little	Agree	Strongly Agree	☐ Prefer not to say
**12.**	**Treatment has been beneficial for other people like me**						
1	2	3	4	5	6	7	☐ Not Applicable
Strongly Disagree	Disagree	Disagree a Little	Neither agree nor disagree	Agree a Little	Agree	Strongly Agree	☐ Prefer not to say
**13.**	**Treatment doesn't work for people like me**						
1	2	3	4	5	6	7	☐ Not Applicable
Strongly Disagree	Disagree	Disagree a Little	Neither agree nor disagree	Agree a Little	Agree	Strongly Agree	☐ Prefer not to say
**14.**	**Staff explained things to me well**						
1	2	3	4	5	6	7	☐ Not Applicable
Strongly Disagree	Disagree	Disagree a Little	Neither agree nor disagree	Agree a Little	Agree	Strongly Agree	☐ Prefer not to say
**15.**	**I would have liked more information about what would happen**						
1	2	3	4	5	6	7	☐ Not Applicable
Strongly Disagree	Disagree	Disagree a Little	Neither agree nor disagree	Agree a Little	Agree	Strongly Agree	☐ Prefer not to say
**16.**	**I had to wait a long time for my first appointment**						
1	2	3	4	5	6	7	☐ Not Applicable
Strongly Disagree	Disagree	Disagree a Little	Neither agree nor disagree	Agree a Little	Agree	Strongly Agree	☐ Prefer not to say
**17.**	**I have to wait a long time between appointments**						
1	2	3	4	5	6	7	☐ Not Applicable

Strongly Disagree	Disagree	Disagree a Little	Neither agree nor disagree	Agree a Little	Agree	Strongly Agree	☐ Prefer not to say
**18.**	**I am motivated to change my substance use**						
1	2	3	4	5	6	7	☐ Not Applicable
Strongly Disagree	Disagree	Disagree a Little	Neither agree nor disagree	Agree a Little	Agree	Strongly Agree	☐ Prefer not to say
**19.**	**I am confident I can change my substance use**						
1	2	3	4	5	6	7	☐ Not Applicable
Strongly Disagree	Disagree	Disagree a Little	Neither agree nor disagree	Agree a Little	Agree	Strongly Agree	☐ Prefer not to say
**20.**	**I feel prepared to take up treatment**						
1	2	3	4	5	6	7	☐ Not Applicable
Strongly Disagree	Disagree	Disagree a Little	Neither agree nor disagree	Agree a Little	Agree	Strongly Agree	☐ Prefer not to say
**21.**	**I have experienced negative effects of substance use**						
1	2	3	4	5	6	7	☐ Not Applicable
Strongly Disagree	Disagree	Disagree a Little	Neither agree nor disagree	Agree a Little	Agree	Strongly Agree	☐ Prefer not to say
**22.**	**I want to make the most of the treatment offered to me**						
1	2	3	4	5	6	7	☐ Not Applicable
Strongly Disagree	Disagree	Disagree a Little	Neither agree nor disagree	Agree a Little	Agree	Strongly Agree	☐ Prefer not to say
**23.**	**Now is the right time for me to change my substance use**						
1	2	3	4	5	6	7	☐ Not Applicable
Strongly Disagree	Disagree	Disagree a Little	Neither agree nor disagree	Agree a Little	Agree	Strongly Agree	☐ Prefer not to say
**24.**	**My family are aware that I have been referred to the treatment service**						
1	2	3	4	5	6	7	☐ Not Applicable

Strongly Disagree	Disagree	Disagree a Little	Neither agree nor disagree	Agree a Little	Agree	Strongly Agree	☐ Prefer not to say
**25.**	**My friends are aware that I have been referred to the treatment service**						
1	2	3	4	5	6	7	☐ Not Applicable
Strongly Disagree	Disagree	Disagree a Little	Neither agree nor disagree	Agree a Little	Agree	Strongly Agree	☐ Prefer not to say
**26.**	**My family are supportive of me attending treatment for substance use**						
1	2	3	4	5	6	7	☐ Not Applicable
Strongly Disagree	Disagree	Disagree a Little	Neither agree nor disagree	Agree a Little	Agree	Strongly Agree	☐ Prefer not to say
**27.**	**My friends are supportive of me attending treatment for substance use**						
1	2	3	4	5	6	7	☐ Not Applicable
Strongly Disagree	Disagree	Disagree a Little	Neither agree nor disagree	Agree a Little	Agree	Strongly Agree	☐ Prefer not to say
**28.**	**I have a lot in common with others attending substance use treatment**						
1	2	3	4	5	6	7	☐ Not Applicable
Strongly Disagree	Disagree	Disagree a Little	Neither agree nor disagree	Agree a Little	Agree	Strongly Agree	☐ Prefer not to say
**29.**	**I’m not like other people who are getting substance use treatment**						
1	2	3	4	5	6	7	☐ Not Applicable
Strongly Disagree	Disagree	Disagree a Little	Neither agree nor disagree	Agree a Little	Agree	Strongly Agree	☐ Prefer not to say
**30.**	**I have a positive view of people seeking treatment for substance use**						
1	2	3	4	5	6	7	☐ Not Applicable
Strongly Disagree	Disagree	Disagree a Little	Neither agree nor disagree	Agree a Little	Agree	Strongly Agree	☐ Prefer not to say
**31.**	**Substance us is part of who I am**						
1	2	3	4	5	6	7	☐ Not Applicable
Strongly Disagree	Disagree	Disagree a Little	Neither agree nor disagree	Agree a Little	Agree	Strongly Agree	☐ Prefer not to say

32.	Substance use is a big part of my life						
1	2	3	4	5	6	7	☐ Not Applicable
Strongly Disagree	Disagree	Disagree a Little	Neither agree nor disagree	Agree a Little	Agree	Strongly Agree	☐ Prefer not to say

33.	Staff at the treatment centre are friendly						
1	2	3	4	5	6	7	☐ Not Applicable
Strongly Disagree	Disagree	Disagree a Little	Neither agree nor disagree	Agree a Little	Agree	Strongly Agree	☐ Prefer not to say

34.	Staff at the treatment centre treat me with respect						
1	2	3	4	5	6	7	☐ Not Applicable
Strongly Disagree	Disagree	Disagree a Little	Neither agree nor disagree	Agree a Little	Agree	Strongly Agree	☐ Prefer not to say

35.	Staff at the treatment centre are easy to talk to						
1	2	3	4	5	6	7	☐ Not Applicable
Strongly Disagree	Disagree	Disagree a Little	Neither agree nor disagree	Agree a Little	Agree	Strongly Agree	☐ Prefer not to say

36.	Staff at the treatment centre are non-judgemental						
1	2	3	4	5	6	7	☐ Not Applicable
Strongly Disagree	Disagree	Disagree a Little	Neither agree nor disagree	Agree a Little	Agree	Strongly Agree	☐ Prefer not to say

37.	I was made to feel welcome when I arrived						
1	2	3	4	5	6	7	☐ Not Applicable
Strongly Disagree	Disagree	Disagree a Little	Neither agree nor disagree	Agree a Little	Agree	Strongly Agree	☐ Prefer not to say

38.	My privacy will be protected						
1	2	3	4	5	6	7	☐ Not Applicable
Strongly Disagree	Disagree	Disagree a Little	Neither agree nor disagree	Agree a Little	Agree	Strongly Agree	☐ Prefer not to say

39.	Things I share with staff during treatment will remain confidential						
1	2	3	4	5	6	7	☐ Not Applicable
Strongly Disagree	Disagree	Disagree a Little	Neither agree nor disagree	Agree a Little	Agree	Strongly Agree	☐ Prefer not to say

40.	I feel I can be honest with staff at the treatment centre						
1	2	3	4	5	6	7	☐ Not Applicable
Strongly Disagree	Disagree	Disagree a Little	Neither agree nor disagree	Agree a Little	Agree	Strongly Agree	☐ Prefer not to say

41.	I have experienced negative effects of substance use						
1	2	3	4	5	6	7	☐ Not Applicable
Strongly Disagree	Disagree	Disagree a Little	Neither agree nor disagree	Agree a Little	Agree	Strongly Agree	☐ Prefer not to say

42.	I feel 'on edge' or anxious at the treatment centre						
1	2	3	4	5	6	7	☐ Not Applicable
Strongly Disagree	Disagree	Disagree a Little	Neither agree nor disagree	Agree a Little	Agree	Strongly Agree	☐ Prefer not to say

43.	I feel relaxed and comfortable at the treatment centre						
1	2	3	4	5	6	7	☐ Not Applicable
Strongly Disagree	Disagree	Disagree a Little	Neither agree nor disagree	Agree a Little	Agree	Strongly Agree	☐ Prefer not to say
